# Impact of elemental composition of particulate matter in the airshed of a University Farm on the local air quality

**DOI:** 10.1016/j.heliyon.2020.e03216

**Published:** 2020-01-31

**Authors:** Bamidele Sunday Fakinle, Odera Bassey Uzodinma, Ebenezer Leke Odekanle, Jacob Ademola Sonibare

**Affiliations:** a Department of Chemical Engineering, Landmark University, Omu-Aran, Nigeria; b Department of Chemical Engineering, Obafemi Awolowo University, Ile-Ife, Nigeria

**Keywords:** Environmental science, Atmospheric science, Atmospheric chemistry, Environmental analysis, Environmental assessment, Environmental hazard, Environmental impact assessment, Particulate matter, Deposition flux, Trace elements, Correlation, Anthropogenic elements

## Abstract

The impact of particulate matter on the ambient air quality of Landmark University Farm was assessed using deposition fluxes of Trace elements (TEs) in the airshed of the farm. Deposition gauges were employed to collect both dry and wet deposition samples of particulate matter between 2018 and 2019. Elemental compositions of particulates collected during the sampling period were analyzed using Energy Dispersive X-ray Fluorescence Spectroscopy (ED-XRF). The deposition fluxes of crustal and anthropogenic trace elements were also determined using standard methods. Results showed that in dry season, iron has the highest mean concentration (3283.61 mg/kg), while chromium has the lowest (0.023 mg/kg). On the other hand, in wet season, silicon and nickel have the highest and lowest mean concentrations of 159.34 mg/kg and 0.01 mg/kg respectively. Although the concentrations of these metals were higher in the dry season than wet season, there was no statistical significant difference between the mean concentrations of the elements measured in each season of the year (p > 0.05). The compositions of some of the elements in the particulate matters were found to be far above the recommended exposure limits prescribed by OSHA. The study concluded that the elemental composition of particulate matter in the airshed of the University Farm adversely impacts the ambient air quality of the Community.

## 1 Introduction

Urbanization, industrialization and other anthropogenic activities have not only caused significant rise in the emissions of trace components, but have also brought about changes in the amount of particles in the air (Bacardit and Camarero, 2009; Hsu et al., 2010). Atmospheric deposition originates from the accumulation and transport of particles onto a surface in the absence of precipitation. Atmospheric deposition does not only determine the fates of airborne toxics but also affects the transfer of material to natural surfaces. Atmospheric deposition is regarded as an important method for the relocation of trace elements, some of which cause serious environmental and human health issues (Galloway et al., 1982; Niu et al., 2015; Pan and Wang, 2015). It has been reported for instance, that atmospheric particles decrease visibility and are also responsible for some respiratory diseases (USEPA, 1996). Furthermore, particulate matters have been described as one of the primary air pollutants containing a wide spectrum of elements with different degree of toxicity and harmfulness (Sherwood, 2002; Sekhavatjou et al., 2010) as well as severe impacts on the respiratory system (Seinfeld and Pandis, 2006), which result in increased morbidity and mortality rates (Gotschi et al., 2005; Valavanidis et al., 2006; Sekhavatjou et al., 2011). Growing concern about particulate matters is due to their toxicity, carcinogenic characteristics (Cao et al., 2009) as well as their continuous presence in the environment (Sherwood, 2002; Hosseini et al., 2012).

The composition and size distribution of particles, and their damaging impacts, are heavily dependent on procedures of particle formation, i.e. particle sources. This has been studied in several PM_10_ and PM_2.5_ research (Michael and Christos, 2006; Tsai et al., 2008; Cheng et al., 2008; Onat and Baktygul, 2013). Traffic, power manufacturing using fossil fuels and biomass, industrial sources, soil re-suspension and marine salt spray are the primary sources of PM, but the comparative contribution of distinct sources differs significantly in moment and place. Quick dust chapters in many regions around the globe cause some of the greatest levels of main ambient PM. These events of dust often demonstrate seasonal concentration patterns which are much more pronounced from most anthropogenic sources than the concentration patterns of PM.

The key problem for risk management is to identify the sources of particulate matters that are accountable for the overall public health burden. A sound understanding of the sources of particulate matter and possible variations in the health impacts of particulate matter could enable sources to be differentiated in terms of their significance to human health and thus allow for more effective risk management. Sources influencing PM levels at a specified place can be determined by using technique of source allocation based on PM chemical compositions, measurement information for air pollution, and meteorological information. Source distribution surveys provide an assessment of the average source impact on local air quality as well as time series information (inconsistency) on source-specific PM levels. The estimated daily PM concentration from each source can then be linked to the daily variability in epidemiological studies in the observed or measured health impacts. Thus, in an epidemiological study, separating particulate matter into sources and analyzing their separate associations with health effects could produce results that could be used directly in risk assessment, risk management, and setting guidelines and standards for air quality. The study seeks to assess the impacts of particulate matters on the ambient air quality of the Landmark University farm. This is with a view to creating awareness and alertness to the health issues of the University community especially as it relates to air pollution. The results obtained in this study will provide a useful databank of particulate matter concentrations within the University community and possibly create a need for effective monitoring of the impacts on both environmental quality and human health.

## 2 Material and methods

### 2.1 Study area

The Landmark University farm is situated within the community of Landmark University in Omu-Aran Local Government Area, Kwara State of Nigeria. It is located at 8º07′54″ N and 5º03′24″ E. It is characterized strictly by agricultural activities ranging from animal production to crop production and it is surrounded basically by vegetated land mass. The farm currently operates on 302 ha of land with activities such as maize, soya bean and vegetable farming. Other activities on the farm include includes fishing and livestock farming.

### 2.2 Sampling

Samples were collected at the study area using fabricated deposition gauges. This equipment is similar to those described by Paode et al. (1998) and subsequently used by Moaref et al. (2014). The deposition gauge was mounted on iron tripod at some distance above the ground at various sampling points to avoid recollection of re-suspended particles. The gauge is made of concrete base to avoid being blown away by wind. The deposition samples were filtered unto a pre-weighed filter paper. The filter paper was weighed after the collection to determine the total mass of the particle collected (Paode et al., 1998). The sampling period was for about 30 days spanning over 6 months each, during both wet and dry seasons. Wet atmospheric depositions were collected within and outside the Landmark University farm. Ten sampling points were selected for this study based on several parameters such as the size of the farm site, facilities present, and locations far from buildings and trees. Eight points were located on the farm site while two points were located in the academic area of the University to serve as the reference or control points.

### 2.3 Analysis

Particles collected by the deposition gauge were analyzed by Energy Dispersive X-ray Fluorescence Spectrometer (ED-XRF) made by Varian Laboratory (EDX-8000VF) and operated at 40 kV and 18 mA. The analysis was carried out at the Center for Energy Research and Development, Afe Babalola University, Ado-Ekiti, Ekiti state, Nigeria. Analytical methods used in this study followed the procedures described by Paode et al. (1998) which was later employed by Yi et al. (2001a,b). Pellets of 19 mm diameter were prepared from the samples by mixing the samples with three drops of organic binder (PVC dissolved in Toluene) and carefully mixed and pressing this in a hydraulic press. The pellets were loaded in the sample chamber of the spectrometer. Maximum voltage of 40 kV and a maximum current of 1mA were applied to produce the X-rays that excite the samples for a preset time (10mins in this case) and in total, 18 trace elements Zn, P, Pb, Al, Cu, Fe, Si, Mn, Ni, Ti, Cr, Co, Cd, K, Na, Ca, Mg, N were detected in the sample.

### 2.4 Quality assurance/quality control

During the sampling and analysis, extreme caution was ensured in order to avoid contamination the samples with metallic laboratory tools such as knives, pincers, ovens etc. During drying, unused filter papers were carefully placed on glass rods to avoid contact with metallic tray. Plastic laboratory wares and distilled water were used throughout the sampling and analysis. Background contamination of the trace elements was routinely monitored using operational blanks that were processed simultaneously with the field samples. At least 10% of the samples were analyzed in duplicate by spiking with a known amount of elements to calculate recovery efficiencies. The recovery efficiencies varied between 85% and 105%.

## 3 Results and discussion

The descriptive statistics results of elemental analysis in dry and wet seasons are presented in Table 1. Although, there was no statistical significant difference between the mean concentrations of the elements measured in each season of the year (p > 0.05), it was generally observed that, the concentrations of the metals were higher in the dry season than in the wet season. This could be due to atmospheric condition such as high wind and temperature experienced in dry season which is believed to favor deposition of particulate matter. Similar observation was reported by Osada et al. (2014). The range of concentrations of Zn, P, Pb, Al, Cu, Fe, Si, Mn, Ni, Ti, Cr, Co, Cd, K, Na, Ca, Mg and N in dry season were 98.9–420.9 mg/kg, 12.5–28.45 mg/kg, 1.48–3.15 mg/kg, 277.56–422.75 mg/kg, 42.9–70.81 mg/kg, 2286–4985.2 mg/kg, 366.3–818.79 mg/kg, 208–632.6 mg/kg, 0.01–0.02 mg/kg, 0.02–0.03 mg/kg, 0.11–0.66 mg/kg, 0.11–0.66 cmol/kg, 0.16–2.08 cmol/kg, 9.14–12.69 cmol/kg, 14–17.2 cmol/kg, 10.96–28.2 cmol/kg, 28.18–132.45 cmol/kg and 30.13–36.44 g/kg respectively. The range of concentrations for the same elements in wet season were 0.13–3.64 mg/kg, 3.55–9.81 mg/kg, 0.09–1.72 mg/kg, 2.26–12.03 mg/kg, 0.09–9.32 mg/kg, 0.18–2.35 mg/kg, 10.56–230.11 mg/kg, 0.17–1.16 mg/kg, 0.01–0.02 mg/kg, 0.01–0.02 mg/kg, 0.01–0.03 mg/kg, 0.01–2.24 mg/kg, 0.09–0.14 cmol/kg, 2.34–4.98 cmol/kg, 0.42–1.06 cmol/kg, 0.66–8.5 cmol/kg, 0.09–0.84 cmol/kg and 0.7–1.83 g/kg respectively.

**Table 1:** Descriptive Statistic of elemental concentrations in dry and wet seasons.

Elements	DRY SEASON					WET SEASON				
	N	Min	Max	Mean	SD	N	Min	Max	Mean	SD
Zn (mg/kg)	8	98.90	420.90	273.52	144.95	7	0.13	3.64	2.54	1.65
P (mg/kg)	8	12.50	28.45	21.56	5.47	7	3.55	9.81	5.46	2.86
Pb (mg/kg)	8	1.48	3.15	2.55	0.59	7	0.09	1.72	1.08	0.66
Al (mg/kg)	8	277.56	422.75	364.09	46.89	7	2.26	12.03	5.55	4.88
Cu (mg/kg)	8	42.90	70.81	54.47	9.92	7	0.09	9.32	1.60	3.41
Fe (mg/kg)	8	2286	4985.2	3283.61	988.37	7	0.18	2.35	1.42	0.91
Si (mg/kg)	8	366.3	818.79	489.23	190.13	7	10.56	230.11	159.34	101.25
Mn (mg/kg)	8	208.5	632.60	412.74	165.91	7	0.17	1.16	0.67	0.44
Ni (mg/kg)	8	0.01	0.02	0.05	0.07	7	0.01	0.02	0.01	0.004
Ti (mg/kg)	8	0.02	0.03	0.024	0.01	7	0.01	0.02	0.02	0.007
Cr (mg/kg)	8	0.02	0.03	0.023	0.01	7	0.01	0.03	0.02	0.006
Co (mg/kg)	8	0.11	0.66	0.45	0.18	7	0.01	2.24	1.51	1.03
Cd (cmol/kg)	8	0.16	2.08	1.46	0.65	7	0.09	0.14	0.09	0.04
K (cmol/kg)	8	9.14	12.69	10.55	1.21	7	2.34	4.98	3.78	1.33
Na (cmol/kg)	8	14.00	17.2	14.96	1.14	7	0.42	1.06	0.77	0.27
Ca (cmol/kg)	8	10.96	28.2	14.35	5.83	7	0.66	8.5	3.50	2.63
Mg (cmol/kg)	8	28.18	132.45	47.68	36.09	7	0.09	0.84	0.50	0.28
Ni (g/kg)	8	30.13	36.44	33.09	3.71	7	0.7	1.83	1.14	0.45

**Figure 1:**
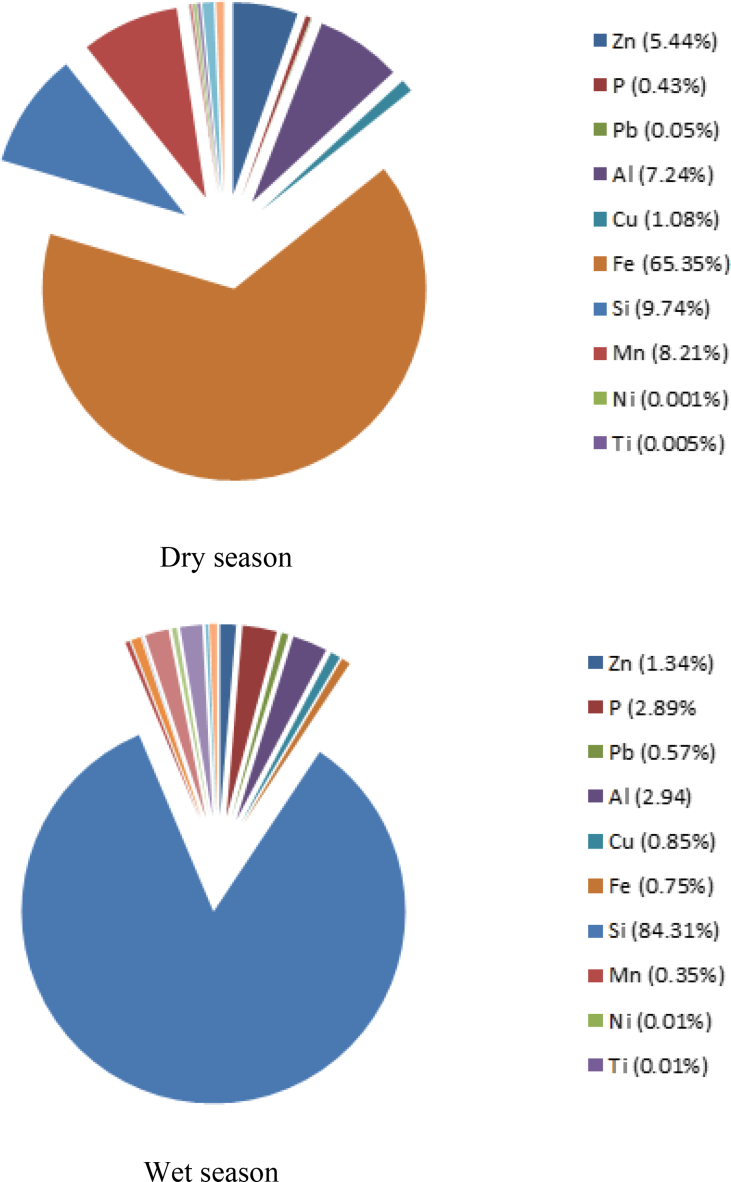
Plot showing the percentage mean elemental concentrations during dry and wet seasons.

Highest mean concentration level was recorded for iron (3283.61 mg/kg) and lowest for chromium (0.023 mg/kg) in dry season while silicon and nickel have the highest and lowest mean concentration of 159.34 mg/kg and 0.01 mg/kg respectively in wet season as shown in Figure 1. Except nickel Manganese and few others whose concentration fell below the regulatory limit of 1000 mg/m^3^ and 5000 mg/m^3^ respectively, as set by the Occupational Safety and Health Administration (OSHA), concentrations of majority of other elements were far above the recommended exposure limits prescribed by the OSHA especially in dry season. For instance, OSHA prescribed that the levels of copper in the ambient air in the workplaces should not exceed 0.1 mg/m^3^ and 1.0 mg/m^3^ for copper dust while lead and zinc should not exceed 0.005 mg/m^3^ and 500 mg/m^3^ respectively. In this study however, the range of the measured concentrations for these elements are 55.4–91.50 mg/m^3^ for copper, 1.91–4.0 mg/m^3^ for lead and 127.8–544.0 mg/m^3^ for zinc in dry season. These values were higher that OSHA permissible limit. Although, the average elemental concentrations in this study were lower than the values reported by Odabasi et al. (2002) but higher than some other previous studies (Moaref et al., 2014; Yi et al., 2001a), the results and observations were comparable to the one measured by Yi et al. (2001b). High concentration of iron recorded is thought to be due to the closeness of the Engineering Workshop of the University (where several metallurgical operations are being carried out on daily basis) to the study site. The results also revealed high concentration of silicon which must have come from the ploughing and other agricultural operations on the farmland. Agricultural activities such as soil tillage and preparation of seed beds, planting, application of fertilizers and pesticides, harvesting and post-harvesting procedures can cause emission of particulate matter. Dry deposition fluxes of the trace elements and the descriptive statistics are presented in Tables 2 and 3 respectively.

**Table 2:** Dry deposition flux of trace elements (mg/m^2^.month) in Landmark University Farms.

	PROPERTIES (mg/m^2^.month)	S1	S2	S3	S4	S5	S6	S7	S8
1	Zinc	0.35	0.33	0.09	0.10	0.25	0.08	0.35	0.24
2	Phosphorus	0.01	0.01	0.03	0.02	0.02	0.02	0.02	0.02
3	Lead	-	-	-	-	-	-	-	-
4	Aluminum	0.27	0.34	0.34	0.30	0.30	0.21	0.35	0.29
5	Copper	0.06	0.06	0.05	0.04	0.03	0.04	0.04	0.04
6	Iron	4.17	3.67	2.08	2.78	1.95	1.95	2.54	2.35
7	Silicon	0.69	0.63	0.38	0.31	0.29	0.28	0.34	0.31
8	Manganese	0.52	0.51	-	0.18	0.19	0.31	0.34	0.30
9	Nickel	-	-	-	-	-	-	-	-
10	Titanium	-	-	-	-	-	-	-	-
11	Chromium	-	-	-	-	-	-	-	-
12	Cobalt	-	-	-	-	-	-	-	-
13	Cadmium	0.68	0.62	0.32	0.05	0.59	0.42	0.48	0.56
14	Potassium	3.39	3.70	4.51	3.44	2.86	2.70	3.30	3.17
15	Sodium	5.61	4.98	5.25	4.37	4.89	4.21	4.62	4.32
16	Calcium	9.20	5.17	4.10	3.60	3.43	3.55	4.04	3.68
17	Magnesium	20.25	41.98	11.42	8.97	7.685	9.651	10.600	11.189
18	Nitrogen	26.19	21.29	33.16	26.23	29.25	27.44	29.57	23.74

**Table 3:** Descriptive Statistics of dry deposition flux.

Properties (mg/m^2^ month)	Min	Max	Mean	Standard Deviation
Zinc	0.08	0.353	0.22	0.121
Phosphorus	0.01	0.03	0.02	0.005
Lead	-	-	-	-
Aluminum	0.21	0.35	0.30	0.046
Copper	0.03	0.06	0.05	0.009
Iron	1.95	4.168	2.69	0.825
Silicon	0.28	0.685	0.40	0.161
Manganese	0.16	0.52	0.33	0.139
Nickel	-	-	-	-
Titanium	-	-	-	-
Chromium	-	-	-	-
Cobalt	-	-	-	-
Cadmium	0.05	0.68	0.46	0.203
Potassium	2.70	4.51	3.38	0.553
Sodium	4.21	5.61	4.78	0.493
Calcium	3.43	9.20	4.59	1.939
Magnesium	7.68	41.98	15.22	11.466
Nitrogen	21.29	29.57	27.11	3.668

The magnitude of the dry deposition flux of the trace elements for each element varied significantly, ranging from 8.360 × 10^−6^ (mg/m^2^.month) for Ni in S1 to 33.161 (mg/m^2^.month) for nitrogen in S3. The highest dry deposition fluxes found were those for nitrogen across all sample points. Elements such as Nickel, titanium and chromium had no dry deposition flux across all sampling points during the study period. Monthly average fluxes of primarily crustal elements (silicon, aluminum, iron, sodium, magnesium, calcium etc.) were higher than the flux of primarily anthropogenic elements (zinc, lead, Copper, Cobalt etc.). This is in agreement with the observations reported by Yunchao et al. (2018). The range of average dry deposition fluxes of Zn, Pb, Cd, and Cu being the representative of anthropogenic elements were 0.076–0.353, 0.001–0.003,0.050–0.676 and 0.034–0.059 mg/m^2^.month respectively, while those of Si, Al, Na, Ca, and Mg being the representative of crustal elements are 0.277–0.685, 0.209–0.345, 4.207–5.0608, 3.431–9.195 and 7.685–41–980 mg/m^2^.month. The monthly average dry deposition fluxes recorded in this study were much lower than those reported by previous studies (Yi et al., 2001b; Brewer and Belzer, 2001). This could be attributed to the proximity of smelting and other industrial facilities that impact the atmosphere of the vicinities where those studies were carried out. However, reports from studies conducted at Tel-Shikmona, Israel and Izmir, Turkey showed higher deposition fluxes of these trace elements (Herut et al., 2001; Odabasi et al., 2002).

**Figure 2:**
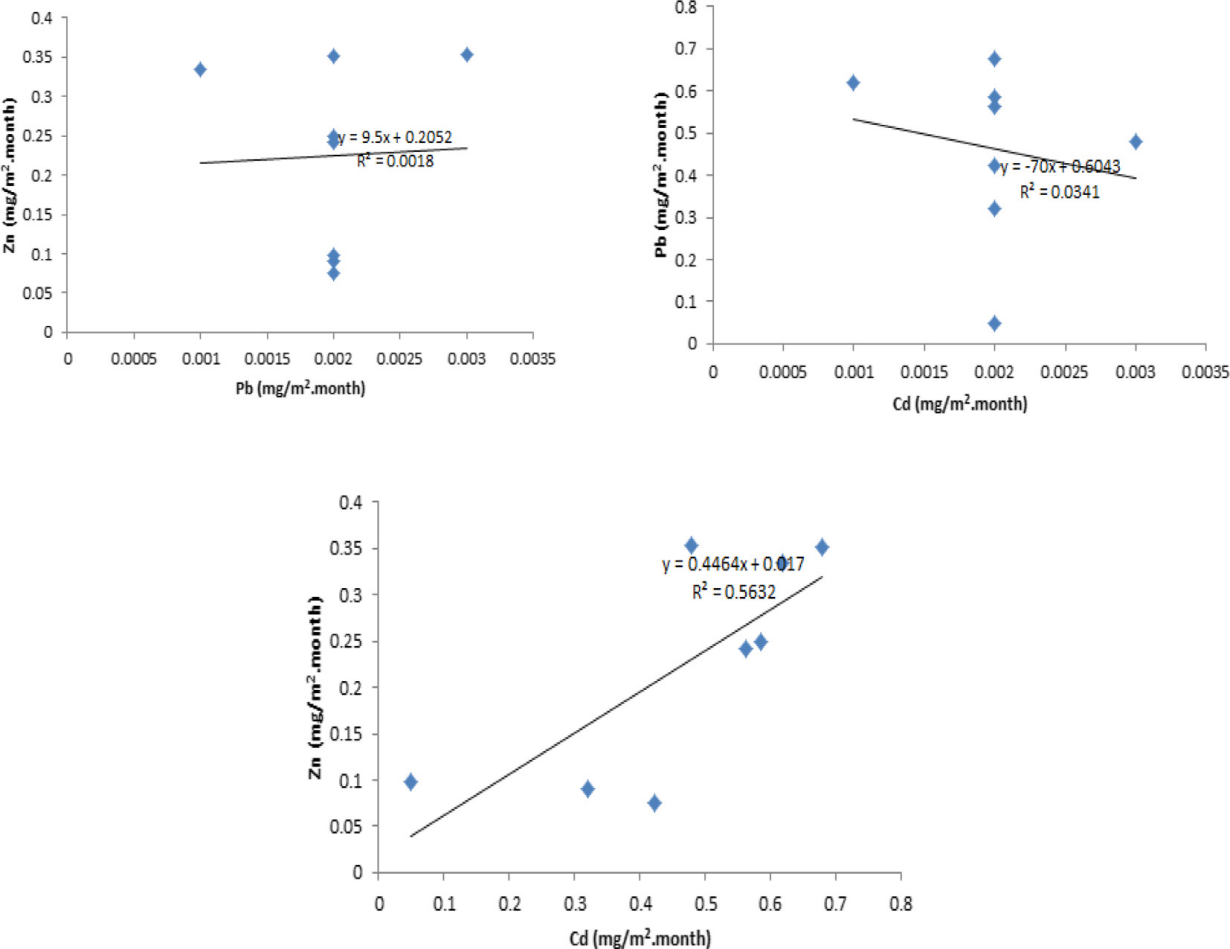
Correlation of heavy metals in dry deposition flux.

Heavy metals are of great importance owing to their toxicities and carcinogenic nature (Yunchao et al., 2018), although they accounted for only a small part of the total deposition flux of the elements. There were significant but weak correlations between Zn and Pb (R^2^ = 0.0018, P < 0.01) as well as between Pb and Cd (R^2^ = 0.0341, P < 0.01). On the other hand, strong significant correlation existed between Zn and Cd (R^2^ = 0.5632, P < 0.01) as shown in Figure 2.

These correlations were in consonance with ones reported by Moaref et al. (2014) who assessed correlations between these heavy metals in the largest industrial city of Iran. Wet deposition fluxes of trace elements are shown in Table 4 while the descriptive statistics results of wet deposition fluxes of trace elements are presented in Table 5. Silicon has the highest wet deposition flux of 9.02031 mg/m^2^.month which was recorded at the gate house of the farm (Sample 4). The deposition flux of nickel was the lowest (0.000392 mg/m^2^.month). The result also showed that asides silicon, wet deposition fluxes of all other trace elements are significantly lower than dry deposition flux. The higher dry deposition flux at the study area could be as a result of the low annual average rainfall of 1500 mm.

**Table 4:** Wet deposition flux of trace elements (mg/m^2^.month) in Landmark University farms.

Properties (mg/m^2^ month)	S1	S2	S3	S4	S5	S6	S7
Zinc	0.13	0.14	0.14	0.14	0.13	0.01	0.01
Phosphorus	0.16	0.14	0.14	0.15	0.15	0.37	0.38
Lead	0.05	0.07	0.05	0.06	0.06	0.00	0.01
Aluminum	0.10	0.10	0.09	0.094	0.10	0.46	0.47
Copper	0.37	0.01	0.00	0.02	0.02	0.01	0.01
Iron	0.05	0.08	0.08	0.08	0.09	0.01	0.01
Silicon	8.33	8.69	8.21	9.02	8.57	0.41	0.48
Manganese	0.02	0.04	0.04	0.03	0.05	0.01	0.01
Nickel	-	-	-	-	-	-	-
Titanium	-	-	-	-	-	-	-
Chromium	-	-	-	-	-	-	-
Cobalt	0.09	0.08	0.08	0.08	0.08	0.00	0.00
Cadmium	0.01	0.00	0.00	0.00	0.00	0.00	0.00
Potassium	0.19	0.11	0.22	0.10	0.20	0.09	0.13
Sodium	0.03	0.03	0.04	0.04	0.04	0.02	0.02
Calcium	0.12	0.14	0.15	0.17	0.33	0.03	0.03
Magnesium	0.03	0.02	0.02	0.02	0.03	0.00	0.01
Nitrogen	0.07	0.07	0.04	0.04	0.04	0.03	0.03

**Table 5:** Descriptive Statistics of wet deposition flux.

Elements	Minimum	Maximum	Mean	Standard Deviation
Zinc	0.01	0.14	0.10	0.064
Phosphorus	0.14	0.38	0.21	0.112
Lead	-	0.07	0.04	0.026
Aluminum	0.09	0.47	0.20	0.180
Copper	-	0.37	0.06	0.133
Iron	0.01	0.09	0.06	0.036
Silicon	0.41	9.02	6.25	3.970
Manganese	0.001	0.05	0.03	0.017
Nickel	-	-	-	-
Titanium	-	-	-	-
Chromium	-	-	-	-
Cobalt	-	0.09	0.06	0.040
Cadmium	-	0.01	-	0.002
Potassium	0.09	0.22	0.15	0.052
Sodium	0.02	0.04	0.03	0.011
Calcium	0.03	0.33	0.14	0.103
Magnesium	-	0.03	0.02	0.011
Nitrogen	0.03	0.07	0.04	0.018

This amount of rainfall could not scavenge aerosol particles hence; high dry deposition flux. Previous studies have also reported similar observations (Yunchao et al., 2018; Yi et al., 2001b). Also, aerosol particle size is one of the important factors that determine the control of the deposition flux of trace elements (Yunchao et al., 2018). Silicon has the highest average flux (6.246296 ± 3.969044 mg/m^2.^month) while nickel has the lowest average flux (0.00047 ± 0.000175 mg/m^2.^month). It should be pointed out that the wet deposition flux in this were significantly lower than those reported in Northern China and Japan (Sakata et al., 2006). The main reason could be attributed to the little or no industrial emission sources in the study area. As presented in Table 5, the ranges of average wet deposition fluxes of Zn, Pb, Cd, and Cu are 0.005096–0.014269, 0.00353–0.06742, 0.00118–0.00549 and 0.00353–0.36534 mg/m^2^.month respectively while those of Si, Al, Na, Ca and Mg are 0.41396–9.02031, 0.08859–0.471576, 0.01646–0.04155, 0.025872–0.3332 and 0.00353–0.03293 mg/m^2^.month respectively.

**Figure 3:**
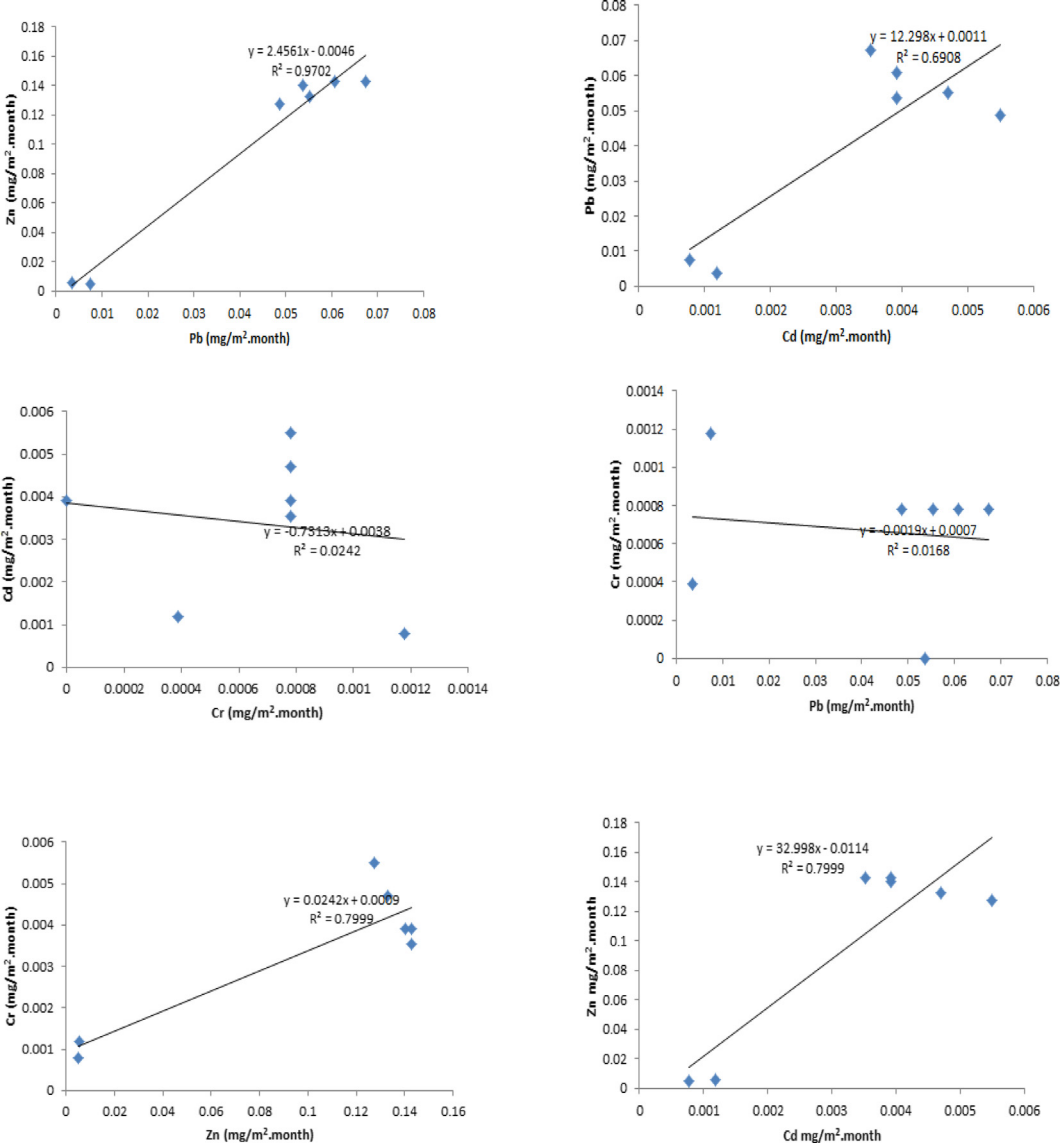
Correlation of heavy metals in wet deposition flux.

Unlike correlations observed in dry deposition flux, there were strong significant correlations between Zn and Pb (r^2^ = 0.9702, P < 0.01) as well as between Pb and Cd (R^2^ = 0.6908, P < 0.01). Significant but weak correlations were observed between Cd and Cr (R^2^ = 0.0242, P < 0.01) and Cr and lead (R^2^ = 0.0168, P < 0.1). According to statistical analysis, it was also observed that there were strong significant correlations between Zn and Cr (R^2^ = 0.7999, P < 0.01) and Cd and Zn (R^2^ = 0.7999, P < 0.01) as presented in Figure 3.

**Figure 4:**
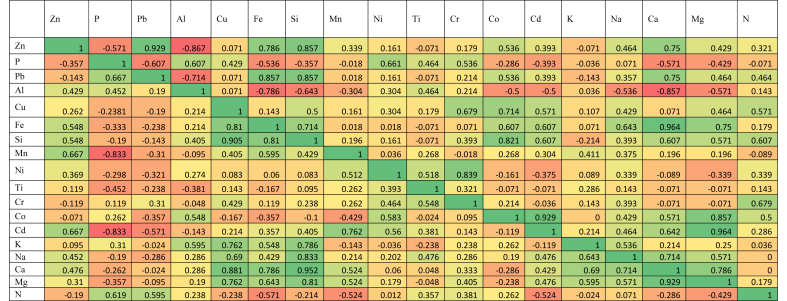
Spearman correlation of dry and wet deposition flux of trace elements.

Using monthly dry and wet deposition fluxes, Spearman Rank Correlation Coefficient (SRCC) was performed to categorize the possible sources of the trace elements (Figure 4). It was observed that there were no correlations between crustal trace elements (Si, Na, Mg, Al and K). On the contrary, there were strong correlations of most anthropogenic elements (zinc, lead, Copper, Cobalt) with Magnesium and Aluminum. This is an indication that Magnesium and aluminum were not really from crustal sources but rather originated from anthropogenic sources. In the wet deposition, good correlations were found between trace elements that are of crustal origin. There were also good correlations between anthropogenic elements (Zn, Fe, Mn, and Cd). However, these elements were not correlated with crustal sources and hence, cannot be said that they originated from crustal sources.

## 4 Conclusion

The study assessed the impacts of particulate maters on the ambient air quality of the Landmark University farms. Elemental compositions of the particulate matter in the airshed of the University Farm were determined. Also, atmospheric wet and dry deposition fluxes of crustal and anthropogenic trace elements were assessed. Correlations of dry and wet deposition fluxes of trace elements were also examined using Spearman Rank Correlation Coefficient (SRCC). Concentrations of the trace elements in dry season were found to be higher than their concentrations in wet season. Apart from this, concentrations of few of the elements fell below the exposure limit set by the Occupational Safety and Health Administration (OSHA), while concentrations of others were far above the recommended exposure limits prescribed by the OSHA and thus, both environmental quality and human health are at risk. Dry deposition fluxes were also found to be significantly higher than wet deposition fluxes due to the low rainfall in Landmark University. It was also observed that concentrations of trace elements and dry deposition flux in locations within the farm were higher than those obtained from most locations outside the University farm. Spearman's correlation revealed good correlations between trace elements that are of crustal origin as well as between anthropogenic elements; which indicate that elements in the same category were from the same source. However, dry deposition and wet deposition fluxes were not correlated. The study concluded that the elemental compositions of particulate matter in the airshed of the University Farm adversely impact the ambient air quality of the Community and hence, control measures are advocated.

## Declarations

### Author contribution statement

Bamidele Sunday Fakinle: Conceived and designed the experiments; Odera Bassey Uzodinma: Performed the experiments; Analyzed and interpreted the data.

Ebenezer Leke Odekanle: Analyzed and interpreted the data; Wrote the paper.

Jacob Ademola Sonibare: Contributed reagents, materials, analysis tools or data.

### Funding statement

This research did not receive any specific grant from funding agencies in the public, commercial, or not-for-profit sectors.

### Competing interest statement

The authors declare no conflict of interest.

### Additional information

No additional information is available for this paper.
